# Clinical Applications of Circulating Tumour Cells and Circulating Tumour DNA in Non-Small Cell Lung Cancer—An Update

**DOI:** 10.3389/fonc.2022.859152

**Published:** 2022-03-15

**Authors:** Joanna Kapeleris, Majid Ebrahimi Warkiani, Arutha Kulasinghe, Ian Vela, Liz Kenny, Rahul Ladwa, Kenneth O’Byrne, Chamindie Punyadeera

**Affiliations:** ^1^ Saliva and Liquid Biopsy Translational Laboratory, The Centre for Biomedical Technologies, The School of Biomedical Sciences, Faculty of Health, Queensland University of Technology, Kelvin Grove, QLD, Australia; ^2^ Translational Research Institute, Brisbane, QLD, Australia; ^3^ School of Biomedical Engineering, University of Technology Sydney, Sydney, NSW, Australia; ^4^ The School of Biomedical Sciences, Faculty of Health, Queensland University of Technology, Brisbane, QLD, Australia; ^5^ Australian Prostate Cancer Research Centre, Queensland, Institute of Health and Biomedical Innovation, Queensland University of Technology, Princess Alexandra Hospital, Translational Research Institute, Brisbane, QLD, Australia; ^6^ Department of Urology, Princess Alexandra Hospital, Woolloongabba, QLD, Australia; ^7^ School of Medicine, University of Queensland, Royal Brisbane and Women’s Hospital, Central Integrated Regional Cancer Service, Queensland Health, Brisbane, QLD, Australia; ^8^ Department of Medical Oncology, Princess Alexandra Hospital, Woolloongabba, QLD, Australia; ^9^ School of Medicine, University of Queensland, Herston, QLD, Australia; ^10^ Saliva and Liquid Biopsy Translational Laboratory, Griffith Institute for Drug Discovery and Menzies Health Institute Queensland, Griffith University, Nathan, QLD, Australia

**Keywords:** lung cancer, NSCLC, circulating tumour DNA (ctDNA), circulating tumour cell (CTC), liquid biopsy

## Abstract

Despite efforts to improve earlier diagnosis of non-small cell lung cancer (NSCLC), most patients present with advanced stage disease, which is often associated with poor survival outcomes with only 15% surviving for 5 years from their diagnosis. Tumour tissue biopsy is the current mainstream for cancer diagnosis and prognosis in many parts of the world. However, due to tumour heterogeneity and accessibility issues, liquid biopsy is emerging as a game changer for both cancer diagnosis and prognosis. Liquid biopsy is the analysis of tumour-derived biomarkers in body fluids, which has remarkable advantages over the use of traditional tumour biopsy. Circulating tumour cells (CTCs) and circulating tumour DNA (ctDNA) are two main derivatives of liquid biopsy. CTC enumeration and molecular analysis enable monitoring of cancer progression, recurrence, and treatment response earlier than traditional biopsy through a minimally invasive liquid biopsy approach. CTC-derived *ex-vivo* cultures are essential to understanding CTC biology and their role in metastasis, provide a means for personalized drug testing, and guide treatment selection. Just like CTCs, ctDNA provides opportunity for screening, monitoring, treatment evaluation, and disease surveillance. We present an updated review highlighting the prognostic and therapeutic significance of CTCs and ctDNA in NSCLC.

## Introduction

Lung cancer is currently the most common cause of cancer-related death worldwide (1) with a total of 1.80 million deaths in 2020 (2). Approximately 80% of lung cancer patients are diagnosed with non-small cell lung cancer (NSCLC), with only 15% surviving for 5 years (3, 4). In the past two decades, an improved understanding of oncogenic driver mutations, such as *EFGR*, *ALK*, and *ROS1*, has led to significant advancements in the treatment of NSCLC patients (5).

Tumour tissue biopsy is still the gold standard for clinical molecular analysis; however, collection of tumour biopsy is invasive and, in most cases, inaccessible due to the location of lung cancer and the ability to safely carry out a biopsy in an elderly patient population with multiple comorbidities. In addition, a comprehensive characterization of different regions of tumour obtained from the same patient has shown intratumour heterogeneity (spatial heterogeneity), as well as differences between serial biopsies over time (temporal heterogeneity) (6). Thus, inter- and intratumour heterogeneity poses a challenge to guide clinical decision as biopsies may be inaccurate in capturing the true genomic landscape of NSCLC. In contrast, the application of the analysis of tumour-derived material in body fluids (liquid biopsy) is currently gaining attention due to its non-invasiveness and the rapid, real-time application in NSCLC that has the potential to overcome tumour heterogeneity (7, 8).

**Figure 1:**
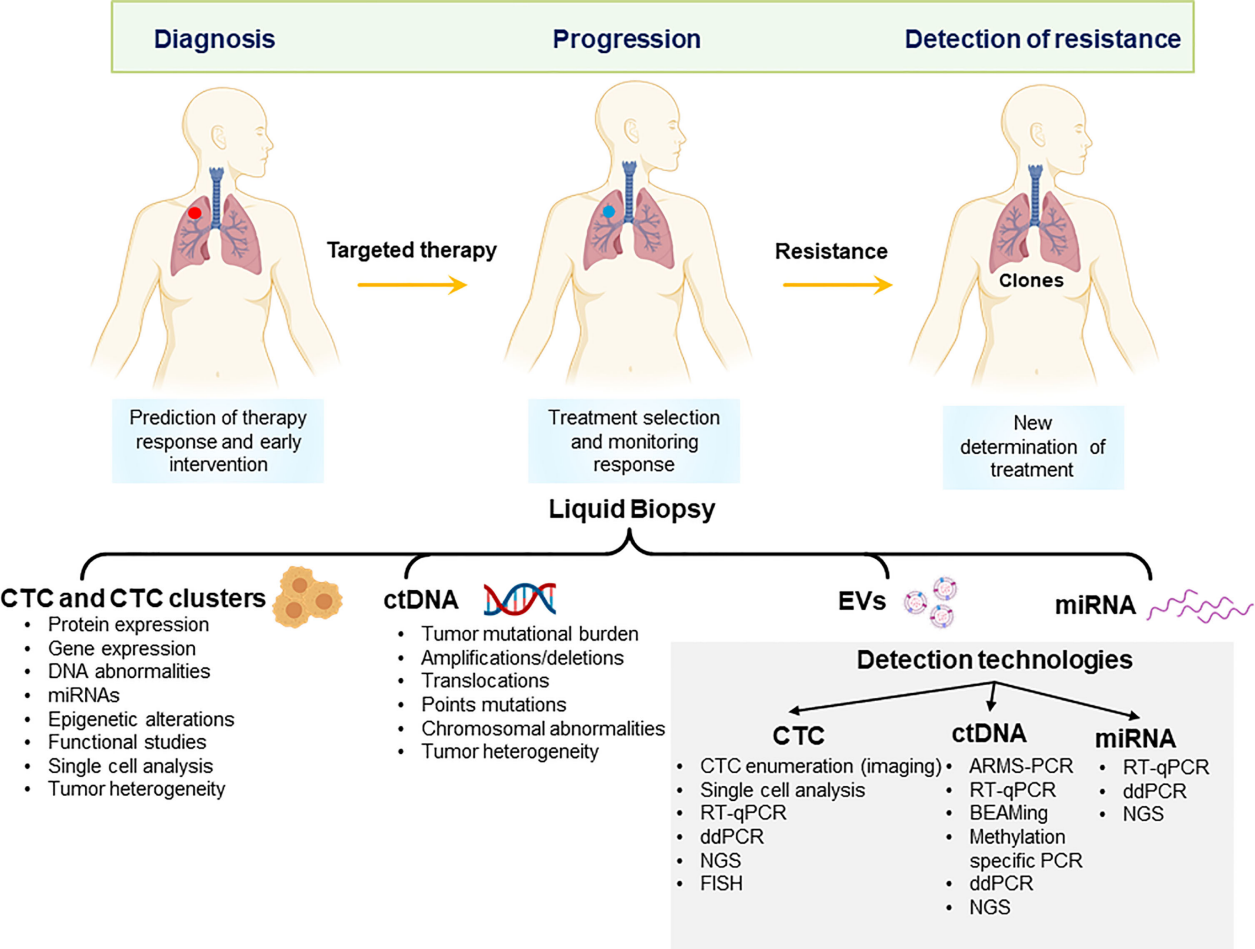
Potential applications of liquid biopsy in the management of non-small cell lung cancer. Liquid biopsy derivatives can be applied to early diagnosis, monitor of response to treatment and detection of resistance to treatment. CTCs, Circulating tumour cells and clusters; ctDNA, circulating tumour DNA; EV, extracellular vesicles; miRNA, microRNA; NGS, next-generation sequencing; ddPCR, digital droplet PCR; FISH, fluorescent *in situ*-hybridization.

The most widely studied liquid biopsy derivatives are circulating tumour cells (CTCs) and circulating tumour DNA (ctDNA). CTCs detach from either primary tumour or metastatic sites and are shed in the patient’s bloodstream, representing a relatively easily obtainable sample of cancer tissue. CTCs were first proposed by an Australian physician Thomas Ashworth in 1869. CTCs are very rare events in the bloodstream; therefore, various enrichment and isolation methods have been developed. The presence of nucleic acids in the circulation was first reported in 1948 by Mandel and Metais (9). Circulating cell-free DNA (cfDNA) is a common derivative found in body fluids such as saliva and blood and is present at a very low concentration (5–10 ng/ml) in healthy individuals (10). cfDNA levels were first demonstrated to be elevated in cancer patients in 1977 (11). While tissue biopsy sampling presents only a snapshot of the tumour at one time or location, the incorporation of CTCs and ctDNA has the potential to overcome tumour spatial and temporal heterogeneity and to provide real-time information relating to tumour burden. This review article highlights the recent advancements in the field of CTCs and ctDNA for the management of patients with NSCLC between 2018 and 2021. Circulating exosomes, microRNA, RNA, and tumour-educated platelets are other appealing tumour derivatives found in body fluids (12, 13) (**Figure 1**).

## Circulating Tumour Cells

Metastasis is a complex, multistage process which requires tumour cells to invade and move from the primary tumour into the circulation, intravasate, survive, extravasate into the bloodstream, and colonize at a distant site leading to a macroscopic metastatic lesion (14). CTCs represent an intermediate stage of metastasis. While rare (estimated to be as low as one to 10 cells per 10 ml of blood), they are uniquely accessible through simple non-invasive sampling of body fluids. To overcome the rarity of CTCs isolated from lung cancer patients’ blood samples, researchers have used pulmonary vein (PV) blood because tumour cells (besides the primary tumour cells) may circulate after passing through the PV (15–17). They found that CTCs were detected in 29 of 30 (96.7%) patients’ PV blood samples.

**Figure 2:**
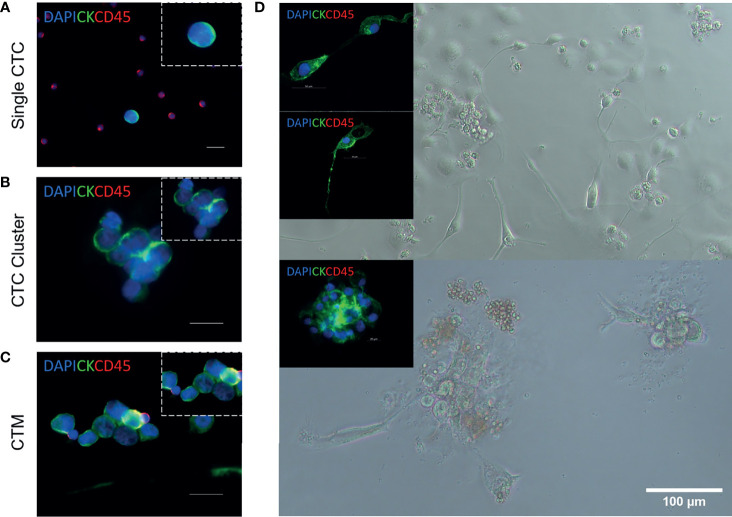
Circulating tumour cell isolation, expansion, and characterization from patients with NSCLC (18). Representative images of single circulating tumour cells (CTCs) **(A)**, CTC clusters **(B)**, and circulating tumour microemboli (CTM) **(C)**. Immunofluorescent staining using anti-CK-FITC, anti-CD45-APC, and DAPI. Scale bar represents 20 μm. **(D)** Isolation, expansion, and characterization of CTCs from patients with advanced-stage NSCLC. Cultured CTCs derived from patient blood samples in a 96-well standard microplate. In-well staining of proliferating cells in culture at day 7. Immunofluorescent staining using anti-CK-FITC and anti-CD45-APC. Cells were shown to be CD45 negative.

CTCs migrate as single cells (**Figure 2A**), clusters (a group of two or more CTCs, **Figure 2B**), and circulating tumour microemboli (CTM) (**Figure 2C**). CTM constitute cellular aggregates, which include platelets, stromal cells, and hematopoietic cells which ‘protect’ the tumour cells from undergoing apoptosis or being attacked by the immune system. As a result, CTM are likely to survive better in the bloodstream (19, 20). CTC clusters and CTM are subject to shear forces in the circulation with some of them having the ability to survive these forces (21). Therefore, they have shown to have a higher metastatic capacity compared to single CTCs (22, 23). In addition, CTCs that have undergone epithelial–mesenchymal transition (EMT) have shown to be associated with a poor survival in NSCLC patients (24–26).

### Circulating Tumour Cell Technologies

Several technologies have been developed to capture CTCs, based on physical or biological properties of CTCs. The CellSearch^®^ system remains the only FDA-approved technology for use in a clinical setting (27, 28) and has been validated as a prognostic and therapeutic tool for monitoring patients with metastatic colorectal (29), breast (28), and prostate cancer (30). CellSearch^®^ is limited to capturing CTCs based on an epithelial phenotype, which is likely to underestimate other clinically relevant CTC phenotypes that have undergone EMT. To address this issue, numerous technologies have emerged as alternative platforms to capture CTCs in circulation. Technologies such as Parsortix, EPIC, RareCyte, and filtration have the potential to overcome the limitations of the CellSearch^®^ system and can be utilized for downstream phenotyping or single-cell analysis (31). Microfluidics-based technologies enable the gentle capturing of CTCs, which then allow for further downstream analysis using cellular, microscopic, or molecular techniques (18, 32–37). Advances in integrated microfluidic platforms made possible the miniaturization of analytical techniques for automated, accurate, and high-throughput assays (38). Moreover, microfluidics presents an opportunity to integrate both isolation and detection in a single device, facilitating the development of true point-of-care diagnostic CTC devices (39). The microfluidic enrichment of CTCs can be utilized through positive [CTC-chip (32), GEDI chip (40)] or negative selection and biophysical properties [iChip (41), MCC (42), CROSS chip (43), spiral microfluidic biochip (44)]. Promising lab-on-a-chip approaches have been extensively reviewed in recent studies (38, 45). One of the drawbacks of microfluidic technology is the challenge of analysing large sample volumes to access key information about rare cells in the circulation.

A large number of isolation and enrichment methods have been used in the past decade with acceptable clinical sensitivity and specificity. However, there is discordance between various CTC isolation and enrichment platforms and cutoff thresholds for CTC enumeration in clinical applications. A study has compared the performance of two CTC detection systems based on the expression of the EpCAM antigen (CellSearch^®^ assay) and on cell size (Isolation by Size of Epithelial Tumour Cells Technology ISET^®^ assay). An underestimation of CTC numbers was determined when using the CellSearch^®^ system in metastatic lung cancer patients (46, 47). A recent study by Papadaki and colleagues compared three CTC enrichment platforms (a. Ficoll density gradient centrifugation; b. ISET^®^, France; and c. Parsortix^®^ technology, ANGLE plc, UK) and showed higher CTC counts when using the Parsortix system. Furthermore, comparison of various CTC enrichment approaches identified variations in CTC capture efficiency, disparities in the enrichment of distinct CTC subpopulations, and discordance in CTC counts (31, 48). Since CTCs are involved in tumour progression, they represent a window to identify mechanisms of metastases. Single CTC profiling may provide better understanding of the origin of tumour, the mechanisms of cancer metastasis, and the study of CTC heterogeneity (49). It is also known that single CTC sequencing and or analysis is important for the advancement of science as bulk analysis may not be representative of the actual tumour and may result in misinformation.

In a recent study by Zeinali et al., CTCs were detected in all 25 NSCLC patients (average of 417 CTC/ml) using a newly developed Labyrinth microfluidic device; they have also identified cells presenting mesenchymal phenotypes in 22 of 23 samples analysed (50). A study by Lim and colleagues was able to predict drug responsiveness in lung cancer patients based on the epithelial–mesenchymal transition (EMT) signature of baseline CTCs using a fluid-based separation FAST disc platform (51). This study highlights the importance of investigating the EMT phenotype on CTCs to investigate the dynamics of disease progression and predict the drug response in NSCLC. A recent study by Zhao et al. profiled the DNA methylome of CTCs from lung cancer patients and identified a unique CTC DNA methylation signature (52). Recent CTC studies in lung cancers are highlighted in **Table 1**.

**Table 1:** Circulating tumour cell studies in lung cancer.

Study	Histology	Sample number	Isolation method	Major findings	Reference
Luojun et al. [2018]	NSCLC	41	Immunomagnetic nanosphere (IMN) separation	A significantly higher CTC capture rate (48.78% *vs*. 73.17%) was obtained. By using a cutoff value of 0 CTC per 2 ml of blood, the sensitivities were 53.66% and 75.61% and the specificities were 100% and 90% for anti-*EpCAM*-MNs or a combination of anti-*EpCAM*-MNs and anti-FRα-MNs, respectively.	(53)
Milano et al. [2018]	NSCLC	10	Immunomagnetic negative depletion	CTCs^EMT^ were detected in three patients at baseline and in six patients after four cycles of cisplatin-based chemotherapy. Longitudinal monitoring of three patients showed that the CTCs^EMT^ detection was associated with poor therapeutic response.	(26)
Turetta et al. [2018]	NSCLC	30	Flow cytometry	*EGFR* and *KRAS* mutations were detected by ddPCR. Mutated DNA was found in 85% of stage IV NSCLC patients, with a 70% match between primary tumour and CTCs. In two patients, multiple *KRAS* mutations were detected. Two patients displayed different mutations compared to the primary tumour, and in two out of four patients with a wild type primary tumour, new mutations were identified: *EGFR p.746_750del* and *KRAS p.G12V.*	(54)
Chinniah et al. [2019]	NSCLC	48	Telomerase-based detection assay	Fifteen out of 20 patients had increased CTC counts in post-radio therapy samples. In 10 out of the 15 patients, CTCs were undetectable on initial post-RT draw but were detected before radiographic detection of occurrence with a median lead time of 6.2 months and mean lead time of 6.1 months between CTC count increase and radiographic evidence of recurrence.	(55)
Li et al. [2019]	NSCLC and SCLC	174 patients 90 controls	Negative enrichment-fluorescence *in situ* hybridization (NE-FISH)	CTCs were detected in 68.29% of patients when the CTC cutoff value was set at 2. The sensitivity of this detection method rose to 82.93% when combining CTC-based detection with measurements of serum tumour markers. Similarly, the sensitivity in patients with stages I–II was improved from 63.93% to 78.69%.	(56)
Lindsay et al. [2019]	NSCLC	550	CellSearch^®^	European pooled analysis examined CTC quantification for prognostication. CTC counts of ≥2 and ≥5 per 7.5 ml were associated with reduced progression-free survival. Survival prediction was significantly improved when incorporating CTC counts to likelihood ratio and clinicopathological models.	(57)
Pailler et al. [2019]	NSCLC	17	Filter laser-capture microdissection, fluorescence-activated cell sorting (FACS), and the DEPArray	Using three platforms, multiple mutations in various genes in *ALK* independent pathways were identified in CTCs of crizotinib-resistant patients. *RTK-KRAS (EGFR*, *KRAS*, *BRAF* genes) and *TP53* pathways were recurrently mutated. In one lorlatinib-resistant patient, two single CTCs out of 12 harboured *ALK* compound mutations, highlighting utility of single-cell sequencing to assess heterogeneity and resistance to *ALK* targeted therapies.	(58)
Scharpenseel et al. [2019]	NSCLC	45	MACS/CellSearch^®^	Enrichment based on either *EGFR* or *HER3* detected CTCs in 37.8% of the patients, while the combination of *EGFR/HER3* enrichment with the *EpCAM*-based CellSearch technique detected a significantly higher number of 66.7% CTC-positive patients.	(59)
Duan et al. [2020]	NSCLC	64	CellCollector^®^	Patients were classified into 4 groups based on their pathological results: benign disease, *in situ* cancer, microinvasive, and invasive. The CTC detection rate for each group was 10.00% (1/10), 45% (5/11), 50% (7/14), and 67% (6/9), respectively. Among patients with lung cancer, the CTC detection rate increased with disease progression. The rate of CTC positivity was 52.94% (18/34) in patients who were diagnosed with lung cancer by pathology and 10% (1/10) in patients with benign disease.	(60)
Frick et al. [2020]	NSCLC	92	Telomerase-based CTC assay	CTCs were detected in 38/92 (41%) subjects prior to stereotactic body radiotherapy (SBRT). A cutoff of ≥5 CTCs/ml before treatment defined favourable (n = 78) and unfavourable (n = 14) prognostic groups. Within 3 months following SBRT, CTCs continued to be detected in 10 of 35 (29%) subjects. Persistent detection of CTCs was associated with increased risk of distant failure (p = 0.04) and trended toward increased regional (p = 0.08) and local failure (p = 0.16).	(61)
Ichimura et al. [2020]	NSCLC	40	Metallic micro-cavity array (MCA) filter	CTC counts were 1.4 ± 0.4, 1.8 ± 1.2, 1.3 ± 0.6, and 7.4 ± 5.1 in clinical stages I, II, III, and IV, respectively. Detection rates (defined as CTC counts of one or more) of each clinical stage were 63.2% (I), 33.3% (II), 66.7% (III), and 71.4% (IV). No significant difference was observed among the stages.	(62)
Katz et al. [2020]	Lung cancer	207	4-colour fluorescence *in situ* hybridization (FISH)	CTCs were detected using FISH probes at *10q22.3/CEP10* and *3p22.1/3q29* in lung cancer cases with 94.2% accuracy, 89% sensitivity, and 100% specificity compared with biopsy.	(63)
Kulasinghe et al. [2020]	NSCLC	20	Spiral microfluidic technology	CTCs were detected in 12/20 NSCLC patients ranging from 1 to 26 CTCs/7.5 ml blood. 3D imaging of CTCs for *ALK* translocations captured a well-defined separation of 3′ and 5′ signals indicative of *ALK* translocations.	(64)
Lim et al. [2020]	NSCLC	40	FAST disc platform	CTCs were identified in 15 samples from 13 patients with mutations consistent with those found in the matching tumour tissue. *EGFR* T790 mutation was detected in both relapsed tissue and CTCs from 2 patients where an initial tumour biopsy did not present this mutation. Analysis of EMT signature of individual pretreatment CTCs is able to predict drug responsiveness in patients.	(51)
Zeinali et al. [2020]	NSCLC	25	Labyrinth device	CTCs were detected in 100% of patients with an average of 417 CTC/ml. Only 31% of CTCs expressed *EpCAM* and among 23 analyzed samples, 22 samples had *Vimentin*+ CTCs. CTC clusters were observed in 96% of patients and correlated with a worse PFS.	(50)
Zhou et al. [2019]	NSCLC	8	Multi-flow microfluidic system	CTCs were detected in 6/8 patients with a median of 12 CTCs/mL and maximum of 78 CTCs/mL	(65)
Huang et al. [2021	NSCLC	36	Subtraction enrichment and immunostaining-fluorescence *in situ* hybridization (SE-iFISH)	After two cycles of cisplatin-based neoadjuvant chemotherapy (NAC), 89% (8/9) of the patients with radiological partial response to NAC had reduced CTC numbers, while 73% (8/11) of the patients with stable disease exhibited increased CTC numbers (p = 0.0098). On pathological examination, 90% (9/10) of patients with a tumour cell necrosis rate (TCNR) lower than 30% had >1 CTC post-NAC, while 80% (4/5) of patients with a TCNR higher than 30% had ≤1 CTC post-NAC (p = 0.017). In aneuploidy analysis, the positive rate (CTC > 0) of triploid CTCs was found to have increased after NAC, in contrast with the tetraploid and multiploid CTCs. Furthermore, tetraploid and multiploid CTCs were found to be significantly downregulated in the patients with partial response to NAC.	(66)
Kong et al. [2021]	NSCLC-adenocarcinoma	16	DropCell platform	Higher degrees of genomic heterogeneity were observed in CTCs compared to ctDNA. Several shared alterations present in CTCs and ctDNA were undetected in the primary tumour, highlighting the intra-tumoural heterogeneity of tumour components that were shed into circulation. Accordingly, CTCs and ctDNA displayed higher degree of concordance with the metastatic tumour than the primary tumour. Alterations detected in circulation correlated with worse survival outcomes. Evolving genetic signatures were detected in the CTCs and ctDNA samples during treatment and disease progression.	(67)
Wan et al. [2021]	NSCLC	48	CellCollector^®^	CTCs were detected in 62.5% (30/48). Using NGS, > 50% of patients had 4 commonly mutated genes, *NOTCH1*, *IGF2*, *EGFR*, and *PTCH1*. 47.37% (9/19) patients had *ARIDH1* mutations. LC-MS untargeted metabolomics found 100 different metabolites, and 10 different metabolites were identified through analysis. This may have potential clinical application value in the diagnosis of CTC-positive early-stage lung cancer (AUC >0.9).	(68)
Wang et al. [2021]	Lung cancer	114	EpCAM immunomagnetic beads	CTCs were detected based on *EpCAM*+ and *CD45*+ cells to exclude white blood cells. In the 98 lung cancer patients, the detection rate of CTCs (≥1 CTC) per 5 ml blood was 87.76%, the number of detections was 1–36, and the median was 2. By sequencing 72 lung cancer-associated genes, a high level of CNVs and gene mutations characteristic of tumour cells were identified.	(69)
Yang et al. [2021]	NSCLC	59	RosetteSep	Utility of hexokinase-2 (HK2) as a metabolic function–associated marker for the detection of CTCs. HK2-based assay identified a novel HK2high/CKneg CTC population with consistent genomic CNV but distinct transcriptome signatures compared to the CKpos counterpart in NSCLC patients. *CK* expression levels are found independent of cellular EMT status in these CTCs and may be related to distinct dissemination mechanisms in different types of body fluids. Selective association of *CK* subtypes in CTCs with patient *EGFR* mutation types may contribute to suboptimal *EGFR* inhibitor therapeutic efficacy in *EGFRL858R* mutant tumours, enabling prediction of patients with poor prognosis before therapy.	(70)
Zhang et al. [2021]	Lung cancer	120	Telomerase reverse transcriptase–based (TERT-based) CTC detection (TBCD)	CTCs based on TBCD can be used as independent biomarkers to distinguish benign from malignant nodules and are significantly superior to serum tumour markers. The detection sensitivity and specificity of CTC diagnosis were 0.854 and 0.839, respectively. For pulmonary nodules ≤1 and 1–2 cm, the sensitivity and specificity of CTCs were both higher than 77%. In addition, the diagnostic ability of CTC-assisted CT was compared by CT detection. CT combined with CTCs could significantly improve the differentiation ability of benign and malignant nodules in lung nodules <2 cm and that the sensitivity and specificity could reach 0.899 and 0.839, respectively.	(71)
Zhao et al. [2021]	NSCLC	15	Negative enrichment	CTC yield was between 10 and 22 CTCs/5 ml of a patient blood sample. DNA methylation patterns were distinct between CTCs and matched primary tumour. Further analysis showed that promoter hypermethylation of epithelial genes is a hallmark of stable EMT.	(52)

### Screening

The majority of lung cancer patients present with advanced disease; therefore, methods to facilitate earlier detection of lung cancer are of high importance. Primary methods of cancer prevention and early detection rely on the practice of screening and are currently available for several tumour types, particularly for colorectal, breast, and prostate cancer (72). Low-dose chest CT (LDCT) is currently used to screen individuals at a high risk of developing lung cancer. LDCT has shown to reduce mortality. However, LDCT as a screening tool has a high false-positive rate (73–75). Blood-based biomarkers might act as a standalone screening tool, refine the selection of patients at risk, or help to classify undetermined nodules detected on LDCT (76). Several published studies have validated CTC isolation using the ISET^®^ technology with high sensitivity and specificity for many cancer types, including NSCLC (46, 47, 76, 77). Ilie et al. in 2014 used the ISET device to diagnose lung cancer nodules earlier in high-risk individuals. CTCs were detected in 3% (5 out of 168 patients) of the patients with chronic obstructive pulmonary disease (COPD) included in the study (76). Furthermore, the annual surveillance of the CTC-positive patients by CT-scan screening was able to detect lung nodules 1 to 4 years after CTC detection, potentially identifying lung cancers at an earlier stage. Interestingly, follow-up of the five patients by CT scan and ISET 12 months after surgery showed no tumour recurrence. In contrast, a prospective, multicentre cohort study by Charles-Hugo Marquette and colleagues enrolled 614 participants from 21 French university centres to test the performance of ISET to capture CTCs. Participants with chronic obstructive pulmonary disease (COPD) had three rounds of screening at 1-year intervals. Subsequently, participants underwent low-dose computed tomography (LDCT) and a matched blood test to detect CTCs using the ISET^®^ platform. The authors concluded that the ISET^®^ was not able to predict lung cancer or extrapulmonary cancer development (78). This is because the ISET^®^ platform showed a low sensitivity to be used as a reliable platform to screen individuals at a higher risk of developing lung cancer. While age, smoking, and gender were comparable to the previous study by Ilie et al. in 2014, the baseline prevalence of lung cancer in the present study was about three times higher than in other studies. This study identified the presence of COPD as an independent risk factor (2–4 times higher in COPD patients than those without COPD) for lung cancer development in patients without clinically detectable lung cancer. The authors also suggest the use of alternative methods for CTC enrichment and detection which may provide better suitability for screening purposes (79). They concluded that a holistic approach of screening integrating clinical, biological, and radiological signatures may be the way forward for a lung cancer screening program.

Frick et al. in 2020 utilized a novel telomerase-based CTC assay and found that patients with elevated counts of CTCs prior to the start of stereotactic body radiotherapy (SBRT), as well as those patients whose CTCs remained persistently detectable after SBRT, were associated with increased regional and distant recurrence. Use of this CTC assay may translate clinically by helping to identify subsets of patients who may maximally benefit from systemic therapy after SBRT for early-stage NSCLC, and to help monitor for tumour recurrence or progression (61).

A study by Duan and colleagues used CTC as a prognostic biomarker to discriminate benign *vs*. malignant nodules as a means of early diagnosing lung cancer. They used a group of 44 patients and subcategorized them based on their pathological results and found CTC detection rates to be increased with the invasiveness of the nodules. While promising, this patient cohort was small and future prospective studies with larger cohorts are needed to confirm the benefits of this technique for detecting early-stage lung cancer nodules, especially in individuals at a higher risk of developing lung cancer (60). Further to this, studies evaluating screening potential of CTCs should also take into consideration the risk of the target population (80). It is difficult to verify that a single blood-based analyte will provide sufficient sensitivity and specificity for lung cancer screening, and therefore, it is likely that a combination approach may overcome the current challenges.

### CTCs as Prognostic Biomarkers

A decrease in CTC counts after surgery and/or chemotherapy may indicate cancer remission while an increase in CTC counts may predict cancer progression. Changes in CTC counts provide important prognostic information (early detection of recurrence or relapse prior to clinical signs) for lung cancer patients and have been associated with poor outcomes with shorter disease-free survival (DFS) (81, 82), progression-free survival (PFS) (83), and overall survival (OS) (7, 84, 85). Recently, a meta-data analysis by Liang and colleagues using five studies involving 351 patients demonstrated the prognostic utility of postoperative liquid biopsy for early tumour recurrence and poor prognosis for DFS in NSCLC patients (86). A recent multicentre European study using the CellSearch^®^ platform using 550 patients demonstrated that CTC counts of ≥2 and ≥ 5 per 7·5 ml are associated with reduced progression-free survival (PFS) and overall survival (OS), respectively (57). They concluded that CTCs can be used as independent prognostic indicators of PFS and OS.

### Monitoring of Response to Treatment Using Circulating Tumour Cells

Monitoring CTC counts during therapy allows assessment of disease development in real time and in some cases may be demonstrated prior to obvious clinical signs of relapse. A decreased CTC count after surgery and/or chemotherapy may indicate cancer remission while an increased count may depict cancer progression. Despite the potential value, clinicians do not utilize CTC information to determine treatment decisions. One of the reasons for this is the lack of a standardized methodology for CTC enrichment and detection, the rarity of CTCs in circulation, and the lack of technology that can be easily integrated into a clinical setting (85, 87). Furthermore, optimal CTC cutoff values vary across isolation methods and clinical trials and therefore require standardization (88). However, there is currently no CTC assay in clinical use for monitoring response to therapy in lung cancer patients due to inconclusive clinical utility data.

In a prospective study with stage IV NSCLC patients (n = 81), 63% of patients initiating therapy had a change in CTC counts and was predictive of survival in patients receiving chemotherapy (89, 90). In a study by Huang et al., an *EpCAM*-independent method of subtraction enrichment and immunostaining-fluorescence *in situ* hybridization (SE-iFISH) (21) was used to enrich and characterize aneuploid CTCs in patients with surgically resectable NSCLC undergoing neoadjuvant chemotherapy (NAC). By monitoring CTCs in real time during the process of NAC, the correlations between CTC enumeration and karyotype with radiological and pathological responses were identified and investigated. The findings conclude that karyotyping aneuploid CTCs can serve as a surrogate marker for disease monitoring in NSCLC (63).

A study by Shishido and colleagues analysed a subset of patients to determine the significance of a high-definition single-cell assay (HD-SCA), to detect CTCs in stage IV NSCLC patients at the initiation of therapy. This non-enrichment-based workflow detects CTCs based on morphology which are identified as high-definition CTCs (HD-CTCs). CTC positivity based on the HD-SCA workflow is inclusive of the cellular morphology of all nucleated cells from the liquid biopsy. Positive HD-CTCs were identified in 51 (62.96%) of patients with a median of 2.20 (range 0–509.20) prior to the start of therapy (90). Patients with an increasing CTC count during the first 3 months post initiation of new treatment had better PFS and OS compared to the other groups. They also found a weak correlation between the absolute number of HD-CTCs at a single time point of therapy and patient outcomes (OS p value = 0.0754).

A case report by Horton et al. presented a 68-year-old male with stage III NSCLC whose primary tumour showed a response to chemoradiotherapy on CT imaging with no evidence of metastatic disease; however, an elevated CTC count was observed post treatment. The patient was later found to have liver metastasis at 3 months on routine imaging. Therefore, an elevated CTC count may have better sensitivity in detecting microscopic residual cancer following curative intent therapy (91).

Despite evidence that monitoring for CTCs during disease progression may provide predictive information (7, 85, 92), its uptake by the clinical community is low. To achieve clinical implementation, standardization of platforms and protocols for CTC isolation and characterization, optimal CTC cutoff values based on the cancer type and stage, and the need for multicentre, large prospective clinical trials are needed in the future (88).

### Therapeutic Implications of CTCs

In the last decade, tyrosine kinase inhibitors (TKIs) have revolutionized the management of NSCLC patients who harbour oncogenic drivers (93). NSCLC, particularly adenocarcinoma, has been separated into molecular subgroups based on oncogenic driver alterations. Epidermal growth factor receptor (*EGFR*)-activating alterations (exon 19 deletion (E19del) and exon 20 insertion mutation) and anaplastic lymphoma kinase (*ALK*) fusion rearrangements are the two most studied driver genes for targeting patients for TKI therapies (94). Additionally, *KRAS* gene mutations and *MET* signalling are widely recognized alterations with important roles in both biological mechanisms and clinical sensitivity to lung cancer treatment (95). First-line *EGFR* mutation inhibitors include gefitinib and erlotinib. They have shown sensitivity in patients harbouring *EGFR*-activating mutations (L858R point mutation in exon 21 and exon 19 deletions). Approximately, 5% of NSCLC patients have these alterations (96). Furthermore, uncommon *EGFR* mutations collectively account for 10% of mutations (exons 18–21) with clinically variable responses to TKI therapies in NSCLC patients (97). Acquired resistance to first-line *EGFR* TKIs is common within 12 months of treatment, mainly due to the T790M mutation, which is detected in approximately 60% of patients (96, 98, 99). Rearrangements in the gene encoding *ALK* commonly involve the echinoderm microtubule-associated protein-like 4 (*EML4*) loci characterizing a unique molecular subgroup (4%) of NSCLC patients (100). *ALK* inhibitors such as crizotinib and alectinib are currently used for ALK-rearranged NSCLC (101). Despite advancements in TKI therapies, acquired resistance occurs in most cases. Limited tumour availability and heterogeneity within the primary site or between primary and metastatic sites are the major hurdles in identifying genetic alterations and selection of patients who are eligible for TKI therapies.

The ability of obtaining tumour cells through a simple blood draw of cancer patients allows for minimally invasive methods to monitor disease progression, treatment selection, and in the case of resistance to tailor treatment modification. Rihawi and colleagues have found the presence of *ALK* rearrangement coupled to *MYC* amplification in tumour and CTCs from the same patient, suggesting a role for *MYC* for primary resistance to crizotinib (102). Furthermore, a study by Pailler et al. highlights the genetic heterogeneity and clinical benefit of CTCs in identifying therapeutic resistance mutations in *ALK*-rearranged patients (103). Several mutations were detected in CTCs of crizotinib-resistant patients including *EGFR*, *KRAS*, *BRAF*, and *TP53* (103). These findings suggest that CTCs provide clinically relevant molecular information and can be used in clinical practice as an alternative to traditional biopsy. Early detection of resistance and identification of acquired mutations in patients undergoing treatment are novel, given that sites of disease can be difficult to access/biopsy and therefore potential resistance mutations may be missed.

### Clinical Significance of *PD-L1* Expression on CTCs

NSCLC patients treated with immune checkpoint inhibitors display durable responses in a subset of patients, but it is currently difficult to predict which patients will benefit from this expensive treatment using current tumour tissue biomarkers, such as the protein expression levels of programmed death ligand 1 (*PD-L1*). *PD-L1* is currently the most commonly used method for predicting response to immune checkpoint inhibitors, but patients who express low or negative *PD-L1* may still benefit from treatment. *PD-L1* expression heterogeneity between primary and metastatic tumours as well as dynamic fluctuations at different time points creates uncertainty in relying on tumour tissue expression of *PD-L1* for treatment selection, particularly for patients whose tumours harbour *EGFR* mutations (104, 105).

Overcoming tumour tissue heterogeneity and difficulties in obtaining longitudinal tumour samples has led researchers to focus on evaluating *PD-L1* expression on CTCs for predictive and real-time monitoring of immune cell activation (106). The presence of *PD-L1*-positive CTCs has shown to correlate with the expression of EMT on CTCs, indicating a partial EMT phenotype (107). Recent studies have been hampered by a small number of patients, and as such data have been inconclusive. Therefore, multicentre prospective trials are required to support the potential clinical utility of *PD-L1* expression levels on CTCs. A summary of the recent studies evaluating *PD-L1* expression on CTCs in lung cancer is depicted in **Table 2**. However, integration of immunotherapies and immune checkpoint blockades targeting either the programmed cell death protein 1 (*PD-1*) or *PD-L1* has been incorporated into routine clinical management of patients with NSCLC and has significantly improved patient outcomes (118–120). *PD-L1* is a dynamic marker that has been shown to switch from negative to positive over the course of chemotherapy/radiotherapy. The increased expression of *PD-L1* during chemotherapy or radiotherapy can be used as a predictor of benefit to immunotherapy, with clinical findings demonstrating efficacy in combining *PD-1/PD-L1* inhibitors with chemotherapy and/or radiotherapy for improved therapeutic outcomes (121). Precision medicine is contributing significantly to improving life expectancy in a subset of patients with advanced NSCLC; however, intra-tumour heterogeneity and acquired resistance are known to significantly impact targeted-agent sensitivity (122). While PD-1/PD-L1 checkpoint inhibitors provide great benefit, randomized studies have known a lack of efficacy for single-agent checkpoint inhibitors (123). The addition of pembrolizumab to standard first-line chemotherapy provided significant survival benefit for EGFR/ALK wild-type patients (124). Evidence suggests that targeted therapy in combination with immunotherapy may benefit as a complementary approach for treatment of NSCLC patients.

**Table 2:** Recent studies evaluating the expression of *PD-L1* on lung cancer-derived circulating tumour cells.

	Study	Histology	Sample number	Isolation method	Major findings	Reference
**Prognostic**	Ilie et al. [2018]	NSCLC	106	ISET^®^ platform; Rarecells	CTCs were detected in 80% of patients, with levels ranging from 2 to 256 CTCs/4 ml. From 71 samples with matched tumour tissue and CTCs, 6 patients (8%) showed ≥1 *PD-L1*-positive CTCs and 11 patients (15%) showed ≥1% *PD-L1*-positive tumour cells in tumour tissue with 93% concordance between tissue and CTCs (sensitivity = 55%; specificity = 100%).	(108)
	Kallergi et al. [2018]	NSCLC	30	ISET^®^ platform; Rarecells	CTCs were detected in 28/30 (93.3%) and 9/11 (81.8%) patients at baseline and after the third chemotherapy cycle, respectively using Giemsa staining. Cytokeratin (*CK*)+/*CD45*- CTCs were detected in 17/30 (56.7%) and 8/11 (72.7%) patients at baseline and after chemotherapy, respectively. At baseline, *PD-1* and *PD-L1* expression levels were observed for 53% and 47% for *CK*+ patients, respectively. After the third treatment cycle, the expression was 13% and 63%, respectively. PFS was significantly shorter in patients with >3 PD-1+ CTCs at baseline as well as patients with Giemsa+ CTCs.	(109)
	Kulasinghe et al. [2018]	NSCLC	56	ClearCell FX	CTCs were isolated in 17/33 (51.5%) of non-small-cell lung cancer (NSCLC) patients. CTCs were determined to be *PD-L1*-positive in 11/17 (64.7%) NSCLC cases. 3D chromosomal DNA FISH for *ALK* and *EGFR* molecular targets showed better resolution than in 2D when imaging CTCs. PFS was not found to be associated with CTCs prior to therapy ([HR]:2.246; 95% [CI]:0.9565–5.273; p = 0.0632), nor the presence of *PD-L1* expression ([HR]:1.646; 95% [CI]:0.5128–5.283; p = 0.4023) in NSCLC patients.	(37)
	Kulasinghe et al. [2019]	NSCLC	35	Spiral microfluidic technology	CTCs/CTC clusters were detected in 26/35 Stage IV NSCLC patients, and subsequently characterized the CTCs for *EGFR* mutation, *ALK* status, and *PD-L1* status.	(35)
	Wang et al. [2019]	NSCLC	13	Graphene oxide (GO) chip	CTCs were detected in 25/38 samples with an average of 4.5 cells/ml. After initiation of radiation therapy, the proportion of *PD-L1*(+) CTCs increased significantly, indicating upregulation of *PD-L1* in tumour cells in response to radiation. In addition, patients positive for *PD-L1* (≥5% of CTCs positive for *PD-L1*) at baseline had shorter PFS. Gene expression analysis showed that higher levels of *PD-L1* were associated with poor prognosis.	(110)
	Cheng et al. [2020]	NSCLC	66	ISET^®^ platform; Rarecells	CTCs were detected in 59 of 66 patients. *PD-L1* positive CTCs were detected in 22 out of the 41 initially treated patients, and 18 of 41 patients showed positive *PD-L1* expression in tumour tissue. The Cohen kappa coefficient of CTC and paired tumour tissue was 0.613. The PFS time of initially treated patients with positive *PD-L1* expression was shorter than for those with negative *PD-L1* expression in CTCs or tumour tissue.	(111)
	Papadaki et al. [2020]	NSCLC	15	Ficoll density gradient centrifugation, ISET, and Parsortix	Ficoll, ISET, and Parsortix presented high yields with phenotypic analysis but provided discordant CTC positivity (13%, 33%, and 60%, respectively) enriching for distinct CTC populations. Indoleamine-2,3-dioxygenase (IDO) and *PD-L1* were expressed in 44% and 33% and co-express in 19%. CTC detection was associated with progressive disease (PD), reduced PFS, and increased risk of relapse.	(48)
	Ntzifa et al. [2021]	NSCLC	30	ISET^®^ platform; Rarecells	*PD-L1* expression was significantly increased at progression of disease compared to the baseline. There was a strong positive correlation between the expression of *VIM* and *PIM-1* at baseline. The high prevalence of *VIM* positive CTCs suggests a dynamic role of EMT during osimertinib treatment; epithelial markers were detected in 37% samples, the expression of mesenchymal/EMT markers (at least one; *VIM*, and/or *TWIST-1*, and/or *AXL*) in 65.4%, and the expression of the stem cell marker *ALDH-1* in 29.6%.	(112)
	Sinoquet et al. [2021]	NSCLC	54	CellSearch	CTCs and *PD-L1*(+) CTCs were detected in 43.4% and 9.4% of patients with NSCLC. *PD-L1* expression concordance between tumour tissue and CTCs was low (54%). The presence of *PD-L1*(+) CTC correlated with the absence of gene alterations in tumour tissue and with poor prognosis-related biological variables. In univariate analysis, absence of gene alterations, number of metastatic sites, prior systemic therapies, and presence of CTCs and *PD-L1*(+) CTCs were associated with worse overall survival, whereas *PD-L1* expression in tumour tissue was not. In multivariate analysis, SCC histology, number of prior systemic treatments, and the presence of CTC were significantly associated with overall survival. Survival was worse in patients with *PD-L1*(+) CTCs compared to patients with *PD-L1*-negative CTC or without any CTC.	(113)
**Predictive**	Dhar et al. [2018]	NSCLC	32	Vortex HT chip	30/31 (96.8%) samples had at least 1 CTC, and 15/31 (48.4%) samples had at least 10 CTCs. Of patient samples with CTCs, 30/31 had one or more *PD-L1* + CTCs.	(114)
	Janning et al. [2019]	NSCLC	127	Parsortix and CellSearch	CTCs were detected in 59 samples using the Parsortix system and 31 samples with CellSearch. CTCs expressing only *PD-L1*+ were identified in 47% of patients, while 47% had *PD-L1*+ and *PD-L1*- CTCs. Additionally, 7% of patients exclusively showed *PD-L1*- CTCs. Upon progression, all patients showed increased *PD-L1*+ CTCs while no change or decrease in *PD-L1*+ CTCs was identified in patients that responded.	(115)
	Koh et al. [2019]	NSCLC and SCLC	67	Microcavity array (MCA) system	CTCs were detected in 66 of 67 patients and more than 5 CTCs were detected in 78% of patients. *PD-L1*-expressing CTCs were detected in 73% of patients, and the proportion score of *PD-L1*-expressing CTCs ranged from 3% to 100%, suggesting intra-patient heterogeneity of *PD-L1* expression on CTCs.	(116)
	Manjunath et al. [2019]	NSCLC	30	Microfiltration system (CellSieve™)	*PD-L1* and EMT markers were expressed at significantly higher proportions in CTCs compared to patient matched tissue. ≥3 *PD-L1* ^pos^/EMT^pos^ CTCs were associated with significantly poorer survival after curative surgical treatment. Expression of *PD-L1* and EMT CTCs was a negative survival predictor for NSCLC.	(25)
	Tamminga et al. [2019]	NSCLC	104	CellSearch^®^	CTCs were detected in 33/104 patients at T0 (baseline) and 17/63 at T1 (4 weeks after treatment). The presence of CTCs at both T0 and T1 was an independent predictive factor for lack of response to checkpoint inhibitors and was associated with worse PFS and OS.	(117)
	Ntzifa et al. [2021]	NSCLC	30	Parsortix	Epithelial and stem cell profile (p = 0.043) and mesenchymal/EMT and stem cell profile (p = 0.014) at progressive disease were correlated. There was a strong positive correlation of *VIM* expression with *PIM-1* expression at baseline and increased *PD-L1* expression levels at disease progression. *AXL* overexpression varied among patients and high levels of PIM-1 transcripts were detected. Expression of *PD-L1* was significantly increased at progressive disease compared to baseline (p = 0.016). The high prevalence of *VIM* positive CTCs suggests a dynamic role of EMT during osimertinib treatment, while increased expression of *PD-L1* at progressive disease suggests a possible prediction for immunotherapy in *EGFR*-mutant NSCLC patients that develop resistance to osimertinib.	(112)

### *Ex-Vivo* Expansion of Circulating Tumour Cells

The expansion of CTCs *ex-vivo* provides a novel disease model to better understand metastasis and to identify drug susceptibility in a preclinical setting (**Figure 2D**). With the inevitable emergence of acquired drug resistance, preclinical models are becoming increasingly novel for individualized treatment. In recent years, several studies have developed methods to propagate CTCs outside of a patient’s body; however, optimal culture conditions are yet to be established. There has been limited success in immortalizing CTC cultures as CTC cell lines long-term, especially for NSCLC (7). Recent studies with successful short-term and long-term expansion are highlighted in **Table 3**.

**Table 3:** Circulating tumour cell cultures derived from blood samples obtained from lung cancer patients.

	Study	Histology	Method of CTC isolation	CTC culture conditions	Group size	CTC lines established	Morphology	Reference
Short-term culture	Balakrishnan et al. [2019]		Centrifugation	After red blood cell (RBC) lysis, cells are cultured on agar microwells in hypoxic conditions (1% O_2_) using high-glucose Dulbecco’s modified Eagle’s medium (DMEM) supplemented with 10% fetal bovine serum (FBS) and 1% penicillin–streptomycin.	52 lung cancer patients			(89)
	Kapeleris et al. [2020]	NSCLC	RosetteSep™	Cultured in a 96-well plate with Han’s medium	70			(18)
	Lee et al. [2020]	SCLC	RosetteSep™	Cells seeded on binary colloidal crystals (BCCs).	22		Three types: large-sized, cohesive round-shaped spheroids, small-sized cohesive irregular or round spheroids and discohesive ‘grape-like’ spheroids	(125)
Long-term culture	Que et al. [2019]	NSCLC	Herringbone-Chip	Cultured in a non-adherent plate with culture medium containing RPMI-1640 medium, epidermal growth factor (EGF), fibroblast growth factor 2 (FGF2), and B27 supplement	109	1	Cells had blebbing surfaces, prominent nucleoli, and high nucleus-to-cytoplasm ratios.	(126)
	Simposon et al. [2020]	SCLC	RosetteSep™/Ficoll xenotransplantation	Xenotransplantation	217	38 CDX	Multiple morphological features of small cell lung cancer (SCLC) were observed, including ‘sheet-like’ cellular architecture (CDX3), pseudorosettes (CDX18), and palisading and trabecular growth (CDX20). Most CDX (35 out of 38) contained neoplastic cells with small nuclei (20–40 μm), consistent with ‘classic’ SCLC. CDX13 and CDX17P had comparatively large nuclei (40–50-μm diameter) consistent with ‘variant’ SCLC morphology. CDX30P had large nuclei. CDX17 exhibited classic morphology suggesting that a switch to the variant morphology of CDX17P occurred during disease progression. CDX29 (limited stage donor) contained cells with classic and variant morphologies.	(127)

Current studies have shown promise in *ex-vivo* cultures of CTCs; however, the success rate is still low, likely due to the small number of isolated CTCs from cancer patients’ blood sample. Higher success rates are observed when CTCs are derived from advanced-stage cancer patients reflecting higher tumour burden. While preclinical models have shown their potential, these models could be complementary to *ex-vivo* CTC cultures. Especially in patients where biopsies are difficult to obtain, CTC-derived explants (CDXs) provide an alternative source. In contrast, patient-derived explants (PDXs) could be utilized where more tumour tissue is available. CDXs can be derived from CTCs collected at different time points during patient follow-up, allowing the generation of paired models that recapitulate the patients’ tumour evolution (128).

With the rising interest in genomic profiling of CTCs to identify driver mutations and possible drug targets to alleviate drug resistance, expansion of CTCs in large quantities will enable identification of novel drug targets and methods for preclinical drug sensitivity testing. The successful short-term expansion of CTCs presents a novel opportunity to test therapies and to conduct functional analysis. However, in recent years, only a few short-term CTC cultures have been reported. Balakrishnan and colleagues cultured CTCs in laser-ablated microwells and reported that *ex vivo*, CTC cluster formation correlates with patients’ response to treatment (89). A recent study by the same authors was successful in expanding short-term CTC cultures from 9 NSCLC patients. They confirmed the presence of lung cancer-specific somatic mutations in CTC cultures using whole exome sequencing (WES) (18).

There has been limited success in long-term CTC cultures derived from NSCLC patients’ blood samples. Que et al. successfully established a CTC cell line from a patient with NSCLC where they tested chemotherapeutic drugs such as docetaxel and cisplatin. The authors identified an increased resistance to their CTCs compared to A549 and 95-D cell lines (126). CDX models bring promise to advancing cancer therapy (129). Expanding CTCs *in vitro* and *in vivo* show promise in better understanding tumour heterogeneity and to predict therapeutic responses; however, clinical trials are needed to validate clinical utility. Future studies should focus on the development of models that consider the tumour microenvironment for a more representative approach.

## Circulating Cell-Free DNA

Like CTCs, the detection of cfDNA provides opportunity for screening, monitoring, treatment evaluation, and disease surveillance (130–134). cfDNA refers to small DNA fragments circulating in the blood that are released from apoptotic and/or necrotic cells (135). Increasing evidence has highlighted the clinical utility of detecting mutations in cfDNA, and the amount of cfDNA in circulation has been correlated with the tumour burden. In addition, cfDNA has been used to detect *EGFR* mutations (exon 19 deletion or exon 21 (L858R) mutations) in selecting NSCLC cancer patients who may benefit from treatment with *EGFR*-TKIs and in identifying drug resistance mutations in lung cancer patients (11, 136–143).

In 2016, the FDA approved the ctDNA assay Cobas^®^ *EGFR* Mutation Test v2 (Roche Diagnostics) for detection of *EGFR* mutations in NSCLC patients as a companion diagnostic to the *EGFR* inhibitor erlotinib. In addition, this year, the FDA also approved the Guardant Health’s Guardant360^®^ companion diagnostic (CDx) liquid biopsy as the first pan-cancer, next-generation sequencing (NGS)-based comprehensive tumour mutation profiling test in patients with any solid cancer tumour. This is also used as a CDx to detect *EGFR* mutations in NSCLC patients who may benefit from treatment with AstraZeneca’s Tagrisso^®^ (osimertinib) (144, 145). The recent TRACERx ongoing multicentre cohort study of 842 patients with NSCLC aims at tracking tumour evolution through longitudinal sampling and sequencing.

The role of ctDNA in molecular diagnosis and disease monitoring was investigated, detecting clonal and subclonal mutations which were present in matched tumour samples (146). The APPLE trial is a multicentre, 3-arm, phase II study evaluating a ctDNA test specific for T790M on a cohort of treatment-naïve *EGFR*-mutant NSCLC patients. The study aims at utilizing ctDNA to identify the optimal approach for sequencing of treatment with gefitinib and osimertinib in advanced NSCLC patients (147). *ROS-1* rearranged in NSCLC, like other oncogene-driven cancers, will eventually develop resistance, highlighting the need for novel *ROS-1* inhibitors (148). Molecular analysis of plasma samples from *ROS-1*-positive NSCLC patients identified seven distinct fusion partners representative of the primary tissue (149). Currently, the most common cfDNA detection platforms are next-generation sequencing and digital droplet PCR (ddPCR) (150–152). Genotyping cfDNA is a fast and accessible possibility to provide insights into tumour heterogeneity and detection of resistance, can allow non-invasive monitoring of disease, and has led to the development of technologies such as droplet ddPCR and NGS. ddPCR and NGS have shown high sensitivity and concordance to detect activating *EGFR* mutations and T790M mutations (153, 154). The NGS-based profiling of NSCLC parents recently has shown robustness in assessing *KRAS* (155) and *ALK* (156) mutational status in circulating DNA and may be valuable in future therapy decision making. The KWAY project assessed the economic sustainability of NGS technology of five Italian referral centres. Results highlighted that the adoption of NGS resulted in reductions of the overall cost of testing per patient (157). NGS enables the analysis of different biomarkers in different patients at the same time and can provide clinically relevant information both before and after targeted treatment, thus assisting treatment decision making in clinical practise (158, 159). Microfluidic-based strategies and lab-on-a-chip (LOC) devices for extraction of nucleic acids provide an opportunity for high-throughput screening with reduced sample volume and rapid quantification (38, 160, 161). An overview of current microfluidic technologies for cfDNA isolation and analysis has been recently reported (162). To demonstrate the clinical utility of ctDNA screening tests, prospective clinical trials comparing early detection strategies based on the ctDNA test with the standard of care are required.

GRAIL is currently conducting a study known as the Circulating Cell-Free Genome Atlas (CCGA) using an NGS approach to develop a reference library of mutations in the blood of patients with common cancers (163, 164). Recently, a novel technique ARMS-PCR has been reported to be highly sensitive and specific, providing a promising approach for the detection of *EGFR* T790 mutation in plasma of cfDNA (165, 166). The use of methylation-specific PCR that profiles epigenetic alterations has also gained attention in recent years. As ctDNA methylation occurs at early stages in lung carcinogenesis, it may assist in early diagnosis (167, 168).

ctDNA analyses provide an advantage with reduced costs and less complex extraction methods of tumour-derived nucleic acids compared to CTCs; however, in some cases, due to the uncertainty of the origin of cfDNA, they may provide inadequate and inaccurate tumour information. The emergence of phenotypic switching in drug resistance *via* non-genetic mechanisms signifies a major obstacle to treatment success (169). CTCs may present a larger research scope than ctDNA as they can maintain the intact genome of living cells and may provide significant information on tumour heterogeneity that ctDNA is unable to provide (130). Krug and colleagues have found higher sensitivity of detecting *EGFR* mutations in plasma when combining exosomal RNA (exoRNA) (98%) and ctDNA (90%) (170). A higher sensitivity for the detection of T790 mutation combining exoRNA and cfDNA (92%) compared to tumour biopsy (89%) confirms the potential clinical utility of liquid biopsy (171).

## Additional Tumour-Derived Biomolecules

Circulating tumour cell-derived biomolecules such as exosomes and microRNA (miRNA) have shown promise as prognostic/predictive biomarkers. Exosomes are a subtype of extracellular vehicles (EVs) comprising nucleic acids, lipids, and metabolites and have been shown to play a role in facilitating tumorigenesis (171). Exosomes are released by all types of cells into extracellular space and once released can act as messengers, gaining traction as potential drug carriers and candidate biomarkers (172).

Circulating RNA molecules in particular miRNA have recently gained attention as potential biomarkers. miRNAs are short non-coding single-stranded RNA molecules (containing about 22 nucleotides) that regulate gene expression at the post-transcriptional level (173). Several studies have shown that circulating miRNAs may reflect tumour biology, with their expression relative to tumour development, progression, and metastases (174). There has been emphasis on their use as a screening tool to predict prognosis and therapy as well as predictors of survival in early-stage patients or patients with metastatic disease (175, 176). The use of exosomes and miRNA show promising results; however, major challenges with variability and lack of standardization have hampered their clinical application. In future, CTCs and ctDNA with the inclusion of additional sources of tumour biomarkers such as exosomes or miRNA may provide a complementary approach, thereby increasing the precision of information obtained through liquid biopsy (**Figure 1**).

## Future Perspective

There is increasing evidence supporting the prognostic utility of CTCs in a number of solid tumour types. CTCs provide a mechanism to investigate tumour biology and to test and develop existing and novel biomarker-driven drugs, leading to precision medicine-based approaches for managing cancer patients (37, 177–180). Clinical implementation of targeted therapies has been hindered due to its accessibility and availability, quality, and quantity of tumour tissue from patients (181). In addition, tumour heterogeneity poses another challenge as a single biopsy may not represent the entire genomic landscape of a patient’s tumour (182). Integration of immune checkpoint inhibitors into clinical practice has presented a new era in immunotherapy; however, only a portion of patients respond to this form of therapy with some patients exhibiting immune-related adverse reactions (183). Recent clinical trials have shown that targeted therapies have improved PFS and OS compared to chemotherapy or immunotherapy (184–187). Identification of predictive biomarkers is an unmet clinical need to stratify patients who may benefit from immunotherapy or combination approaches.

Isolation and enrichment of CTCs remains as one of the main challenges that need to be addressed before CTC workflow can be implemented in a clinical setting (188). Inconsistencies between CTC data across similar patient cohorts and multiple CTC detection platforms, as well as the difficulty in reproducing CTC studies, hinder the translation of CTC into clinics (189). Successful *ex-vivo* short-term cultures of CTCs and long-term establishment of CTC cell lines provide a powerful tool to model tumour heterogeneity *in vitro* at clinically relevant time points and enable preclinical personalized drug testing. Furthermore, future application of CTCs may assist patients through the entire course of disease from diagnosis through to treatment selection, monitoring, and follow-up assessment. A recent meta-analysis evaluated the diagnostic accuracy for CTCs for the clinical determination of lung cancer assessment in 21 studies with 3,997 subjects. The pooled sensitivity and specificity were 0.72 (95% CI: 0.65–0.79) and 0.96 (95% CI: 0.91–0.98), respectively, and the pooled positive and negative likelihood ratios were 16.86 (95% CI: 7.65–37.12) and 0.29 (95% CI: 0.23–0.37), respectively. Zhao and colleagues concluded that CTCs had good diagnostic value for detecting lung cancer (190).

The involvement of ctDNA mutation data to predict acquired treatment resistance and to facilitate therapeutic decision making is becoming increasingly popular (191, 192). This is due to its ease of use, analysis being less expensive, addressing of intra-tumour heterogeneity, and high sensitivity for detecting tumour burden potential. As an example, the androgen receptor splice variant 7 (*AR-V7*) CTC liquid biopsy test by Epic Sciences is the first clinically validated test for castration-resistant prostate cancer (mCRPC) (193). Currently, no similar tests exist for NSCLC.

A recent meta-data study analysed the diagnostic utility of both CTCs and ctDNA for number of gene mutations in lung cancer, highlighting improved diagnostic performance compared to ctDNA analysis alone (150). While CTCs hold promise as prognostic and predictive biomarkers for the management of patients with lung cancer, the lack of standardised CTC isolation and enrichment platforms, the discrepancy of available data, and the lack of agreed cutoff levels make CTCs more difficult to be integrated into clinical practise (194). Future focus on harmonization of studies and data sets may improve the diagnostic and prognostic utility of CTCs (195). Public–private partnerships such as CANCER-ID (https://www.cancer-id.eu/) and the US-based Blood Profiling Atlas in Cancer (BloodPAC) consortium (https://www.bloodpac.org/) are focused on standardizing methods and technologies for circulating blood-based biomarkers. The integration of a complementary approach using additional liquid biopsy derivatives such as ctDNA and exosomes may provide a more comprehensive representation of the tumour genomic landscape, thereby overcoming current challenges with tumour heterogeneity and identifying new targetable mutations (130). Large sample, multicentre cohort, and prospective clinical trials will advance the current understanding of tumour evolution and its effect on cancer biology and patient outcomes.

## Author Contributions

Idea: JK, KO’B, CP. Preparation of figures and tables: JK, ME. All authors were involved in the preparation, review, and editing of the manuscript. All authors contributed to the article and approved the submitted version.

## Funding

CP is funded by Cancer Australia (APP 1145657), a NHMRC Ideas Grant (APP 2002576), and the Royal Brisbane Women’s Hospital Foundation and National Institute of Health.

## Conflict of Interest

The authors declare that the research was conducted in the absence of any commercial or financial relationships that could be construed as a potential conflict of interest.

## Publisher’s Note

All claims expressed in this article are solely those of the authors and do not necessarily represent those of their affiliated organizations, or those of the publisher, the editors and the reviewers. Any product that may be evaluated in this article, or claim that may be made by its manufacturer, is not guaranteed or endorsed by the publisher.
